# Genetically Determined Metabolites in Graves Disease: Insight From a Mendelian Randomization Study

**DOI:** 10.1210/jendso/bvad149

**Published:** 2023-11-30

**Authors:** Yao Tan, Jiayang Yin, Jiamin Cao, Bingyu Xie, Feng Zhang, Wei Xiong

**Affiliations:** Department of Ophthalmology, The Third Xiangya Hospital, Central South University, Changsha City 410013, China; Postdoctoral Station of Clinical Medicine, The Third Xiangya Hospital, Central South University, Changsha City 410013, China; Department of Ophthalmology, The Third Xiangya Hospital, Central South University, Changsha City 410013, China; Department of Ophthalmology, The Third Xiangya Hospital, Central South University, Changsha City 410013, China; Department of Ophthalmology, The Third Xiangya Hospital, Central South University, Changsha City 410013, China; Department of Ophthalmology, The Third Xiangya Hospital, Central South University, Changsha City 410013, China; Department of Ophthalmology, The Third Xiangya Hospital, Central South University, Changsha City 410013, China

**Keywords:** Graves disease (GD), metabolites, Mendelian randomization, LDSC, causal associations

## Abstract

**Context:**

Graves disease (GD) is a prevalent autoimmune disorder with a complex etiology. The association between serum metabolites and GD remains partially understood.

**Objective:**

This study aimed to elucidate the causal connections between serum metabolites and predisposition to GD, examining potential genetic interplay.

**Methods:**

A 1-sample Mendelian randomization (MR) study was conducted on the GD analysis that included 2836 cases and 374 441 controls. We utilized genome-wide association study summary data from the FinnGen project, analyzing the causal impact of 486 serum metabolites on GD. Approaches used were the inverse variance weighted methodology, Cochran’s Q test, MR-Egger regression, MR-PRESSO, Steiger test, and linkage disequilibrium score regression analyses to assess genetic influence on metabolites and GD.

**Results:**

19 metabolites were identified as having a pronounced association with GD risk, of which 10 maintained noteworthy correlations after stringent sensitivity assessments. Three metabolites exhibited significant heritability: kynurenine (OR 3.851, *P* = 6.09 × 10^−4^), a risk factor; glycerol 2-phosphate (OR 0.549, *P* = 3.58 × 10^−2^) and 4-androsten-3beta,17beta-diol disulfate 2 (OR 0.461, *P* = 1.34 × 10^−2^) were recognized as protective factors against GD. Crucially, all 3 exhibited no shared genetic interrelation with GD, further substantiating their potential causal significance in the disease.

**Conclusion:**

This study unveils pivotal insights into the intricate relationships between serum metabolites and GD risk. By identifying specific risk and protective factors, it opens avenues for more precise disease understanding and management. The findings underline the importance of integrating genomics with metabolomics to fathom the multifaceted nature of GD.

Graves disease (GD), an autoimmune thyroid disorder, ranks as one of the most common causes of hyperthyroidism worldwide [1]. The etiology of GD is multifaceted, involving genetic predisposition, environmental factors, and immune system dysfunction [2-4]. Although the exact etiology remains elusive, the autoimmune foundation of the disease is characterized by the infiltration of the thyroid gland by immune cells and the production of autoantibodies against thyroid-specific antigens [5]. Such immune dysregulation culminates in hyperthyroidism and its myriad clinical manifestations, from heat intolerance and weight loss to cardiovascular complications and exophthalmos [1]. Despite substantial research, the complexity of the underlying mechanisms of GD warrants a more nuanced exploration, particularly concerning metabolic changes.

Recent advancements in metabolomics have opened new vistas in understanding various diseases, including GD [6-8]. Serum metabolomics provides a snapshot of an organism's metabolic state, reflecting intricate interactions between genetic, epigenetic, and environmental factors [9, 10]. Analyzing the serum metabolome of GD can uncover key metabolites and pathways associated with disease onset and progression [11-13]. These findings may shed light on disease mechanisms, lead to novel diagnostic markers, or even therapeutic targets. The interplay between metabolic alterations and GD is gradually being unraveled, paving the way for more personalized and targeted therapeutic interventions.

In the study of the intricate connection of GD with metabolomics, Mendelian randomization (MR) and linkage disequilibrium score regression (LDSC) analyses offer promising advantages. MR utilizes genetic instruments as proxies for exposures (eg, metabolites), thus mitigating confounding and enabling causal inference between metabolites and GD [14]. This approach leads to robust insights into the relationships between serum metabolites and the risk of GD, uncovering potentially causative metabolites. LDSC further fortifies the genetic correlation analyses, enabling the exploration of genetic heritability and shared genetic origins [15, 16]. The integration of these methods offers a groundbreaking approach in the field, enhancing our understanding of the complexity of GD, and informing potential preventative and therapeutic strategies.

## Materials and Methods

### Metabolite Data Source Overview

**Figure 1.**
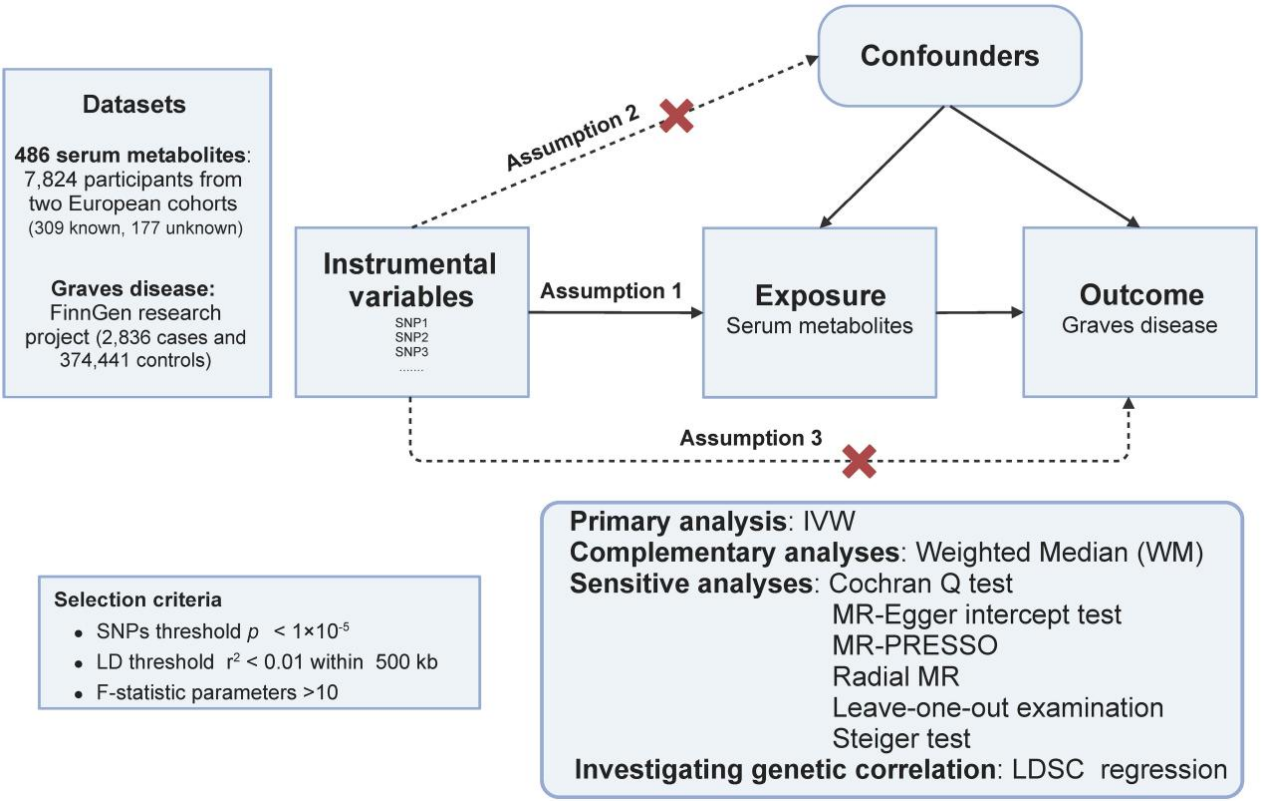
A synoptic depiction of the research workflow.

Our MR investigation, outlined in Fig. 1 and conforming to the STROBE-MR checklist [17], utilized genetic data of serum metabolites from the metabolomics genome-wide association study (GWAS) server. This research was significantly influenced by the extensive work of Shin et al, conducting a nontargeted metabolomics GWAS, thereby pinpointing genetic determinants of 486 serum metabolites [18]. The investigation encompassed 7824 participants from 2 European cohorts: the KORA F4 study in Germany and the UK Twin Study. Informed consent was granted by all participants, with both studies receiving approval from their respective local ethics committees. Fasting serum underwent nontargeted mass spectrometry scrutiny, with standardized metabolic analyses carried out by Metabolon, Inc. Following rigorous quality control, 486 metabolites (309 identified, 177 unidentified) were assessed. These metabolites were classified into 8 biochemical categories in alignment with the Kyoto Encyclopedia of Genes and Genomes (KEGG) database [19]. Approximately 2.1 million single nucleotide polymorphisms (SNPs) were incorporated in the GWAS meta-analysis, with the genotyping details for both cohorts presented in previous studies [18, 20]. All 486 metabolites are shown elsewhere (Table S1 [21]).

### Outcome Sources of GD

Summary statistics for GD GWAS were obtained from the FinnGen research project (https://r9.finngen.fi/) [22]. GD was classified based on the International Classification of Disease, Ninth Revision (ICD-9; 242.0) and ICD-10 (E05.0) codes. The GD analysis included 2836 cases and 374 441 controls, with adjustments made for variables such as age, sex, genetic relatedness, genotyping batch, and the first 10 principal components.

### Selection and Validation of Instrumental Variables

Our study meticulously adhered to 3 foundational assumptions in the selection of instrumental variables (IVs) for MR analysis: (1) pertinence to the metabolite exposure, (2) association with the outcome exclusively through the exposure pathway, and (3) absence of correlation with any confounding variables. To account for potential confounding factors, we utilized the “phenoscanner” R package (version 1.0). Genetic variants corresponding to the 486 metabolites were initially isolated with a significance threshold fixed at *P* < 1 × 10^−5^. Further refinement of independent variants implemented a clumping procedure, establishing a linkage-disequilibrium threshold of r^2^ < 0.01 within a 500 kb proximity. A subsequent filtration process eliminated palindromic SNPs possessing middle allele frequency and SNPs manifesting inconsistent allelic effects (eg, A/G vs A/C). To validate the efficacy of the IVs, we engaged the variance explained (R^2^) and F-statistic parameters, maintaining a minimum constraint of F > 10. The choice of instruments also factored in the mitigation of bias, discarding variants demonstrating inferior statistical robustness and excluding outcome-related SNPs from the IVs (*P* < 1 × 10^−5^). In fulfillment of assumption (3), IVs were diligently pruned to encompass only those affiliated with metabolites and devoid of direct links to the outcome (*P* < 1 × 10^−5^). Ultimately, metabolites correlated with more than 4 SNPs were advanced for MR scrutiny. This exhaustive methodology facilitated the rigorous validation of IVs, bolstering the credibility of our MR investigation.

### MR Analysis

In our inquiry, we harnessed a sophisticated 2-sample MR methodology, focusing on metabolites characterized by at least 4 independent IVs. This methodological stance facilitated statistical exploration and calibration for potential pleiotropy. The cardinal analysis leveraged the multiplicative random-effect inverse variance weighted (IVW) method, culminating in a meta-analysis that amalgamated Wald estimates derived from each SNP, thereby synthesizing a holistic effect estimate of each metabolite on GD [14]. To bolster the credibility of these determinations, we employed the weighted median algorithm. This technique, resilient against up to 50% of invalid SNPs, was invoked for metabolites displaying noteworthy IVW estimates (*P* < .05), thus reinforcing result robustness [23]. Using the previously described MR power calculation method [24], we assessed the statistical power of our study to detect the causal effect of serum metabolites on GD.

### Sensitivity Analysis

The role of sensitivity analysis was paramount in assessing the detrimental effects of horizontal pleiotropy and heterogeneity on our MR estimates. To counter these phenomena, we harnessed a suite of assessments: the Cochran Q test served as a tool for heterogeneity detection [25], the MR-Egger intercept test identified directional pleiotropy and biases [26], and the MR-PRESSO [27] and radial MR [28] techniques were engaged to isolate and rectify outliers and heterogeneous SNPs. Thereafter, we executed a leave-1-out examination to ascertain that no individual SNP exerted a disproportionate influence on our MR estimates [29]. Moreover, to scrutinize the directionality of causality and evaluate the potential influence of GD phenotypes on candidate metabolites, reverse MR analysis and Steiger test were implemented [30]. This procedure assists in obliterating the bias of reverse causality, thereby fortifying our comprehension of the direction and magnitude of causal associations.

Thus, the causal impact of blood metabolites on GD was verified through a rigorous procedure involving (1) a significant *P* value from the primary IVW-derived analysis (*P* < .05), (2) the nondetection of heterogeneity or horizontal pleiotropy, and (3) an absence of marked alterations in MR estimates attributable to a single SNP.

### Investigating Genetic Correlation

To account for any possible genetic correlation between exposure and outcome variables, which could potentially skew our MR estimations, we instigated supplementary analyses [31, 32]. Despite the exclusion of GD associated SNPs during the selection of IVs, unrelated SNPs could inadvertently influence GD genetics. In response to this, we utilized LDSC regression. This tool estimates the co-inheritance of 2 traits via SNP-grounded chi-squared statistics, thereby ensuring that any discerned causal effects are devoid of confounding by coheritability [16]. All statistical analyses were conducted in R version 4.1.0.

## Results

### Instrumental Variables Strength and Validity Evaluation

Within our investigation, we embarked on a 2-sample MR analysis to probe the causal impact of 486 serum metabolites on GD, drawing upon GWAS summary data harvested from the FinnGen project. Among these metabolites, 6 exhibited fewer than 4 IVs (namely fructose, xanthione, glutamate, ergothioneine, X-11843, X-12776) and were thus omitted from our analytical purview. Consequently, we engineered IVs for the residual 480 metabolites, with the enumeration of SNPs fluctuating between 4 and 477 for each individual metabolite (Table S1 [21]). These IVs encompassed a variance of 0.002% to 0.708% within their respective metabolites. Furthermore, the F-statistics associated with all SNPs in relation to metabolites surpassed 10, denoting a robust potency of the IVs.

### Unraveling the Metabolite Links to GD Causality

**Figure 2.**
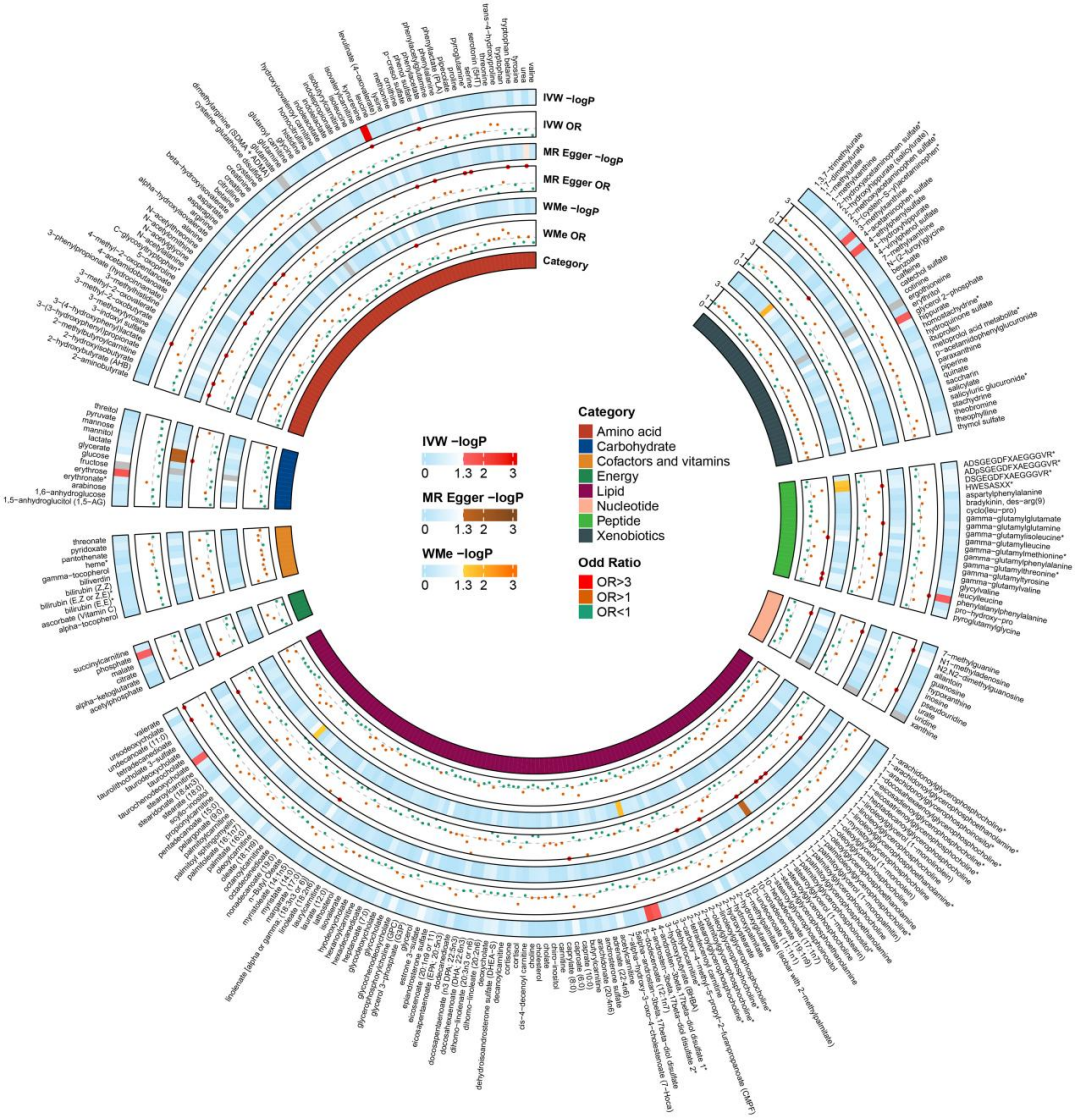
A cyclic heat map visually delineates the causal relationships between serum metabolites and Graves disease. In this configuration, each band corresponds to a specific metabolite. Predominant branches denote distinct metabolite categories, with each being showcased in its own segment. Sub-branches nested within these primary divisions represent the results from varied MR analysis techniques, ranging outward to inward: IVW, MR-Egger, and WMe. Notably, 4 metabolites (fructose, xanthine, glutamate, and ergothioneine) are shaded in gray to signify their exclusion, attributed to their possession of fewer than 4 instrumental variables. IVW, inverse variance weighted; WMe, weighted median; OR, odds ratio; *P*, *P* value.

By employing the IVW method, we delved into the complex causal relationships among 480 metabolites and GD, leveraging GWAS summary statistics. Our rigorous analysis yielded 19 robust causal association features with *P* < .05, comprising 10 identified metabolites and 9 metabolites of indeterminate provenance (Fig. 2, Table S2 [21]). In the succeeding stages of our study, our focus was strategically narrowed to these 10 recognized metabolites. While the IVW method is compellingly effective in deducing causal associations between an exposure and a resultant disease, it may be predisposed to the influence of weak instrument bias. Thus, to enhance the validity of our causal inferences, we conducted supplementary analyses, wherein the application of Cochran’s Q test and MR-Egger regression revealed no significant heterogeneity or horizontal pleiotropy among these known metabolites (*P* > .05).

Following this, we utilized the MR-PRESSO analysis to delve deeper into heterogeneity (Table S3 [21]). Notably, no outliers were detected in the known metabolites (*P* > .05), a finding that is in consonance with earlier results from Cochran’s Q test and MR-Egger regression. Additionally, Steiger testing ascertained that reverse causality did not infringe upon the causal linkage between the genetically determined metabolites and GD (*P* < .05). In the reverse MR analysis, we utilized GD-associated SNPs to assess the potential influence of GD on serum metabolite levels (Table S4 [21]). Additionally, the causal effects determined through this reverse MR analysis between GD and serum metabolites are elaborated elsewhere (Table S5 [21]). The absence of a reciprocal causal impact in this analysis further accentuates the directionality and robustness of our primary results, underscoring the causative relationship of certain metabolites with GD risk (*P* > .05).

### Exploring the Specific Metabolite Correlations in GD

**Figure 3.**
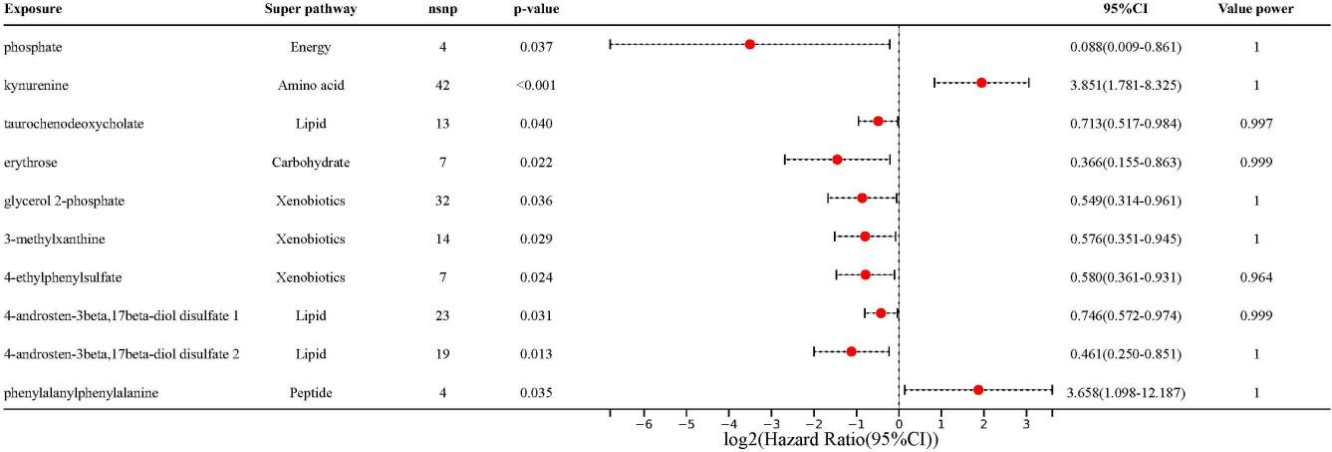
Forest plots elucidating the effect magnitudes of recognized candidate metabolite associations with Graves disease, enhanced by unbroken lines depicting the MR odds ratio and the 95% CI.

Upon carefully eliminating the impacts of horizontal pleiotropy and heterogeneity, we uncovered distinct associations between 10 particular metabolites and the risk of GD, employing the IVW method (Fig. 3). In the energy pathway, phosphate was shown to be a protective factor (OR 0.088, 95% CI = 0.009-0.861, *P* = 3.67 × 10^−2^). Among amino acids, kynurenine was identified as a risk factor (OR 3.851, 95% CI 1.781-8.325, *P* = 6.09 × 10^−4^). Lipids such as taurochenodeoxycholate (OR 0.713, 95% CI 0.517-0.984, *P* = 3.97 × 10^−2^), 4-androsten-3beta,17beta-diol disulfate 1 (OR 0.746, 95% CI 0.572-0.974, *P* = 3.13 × 10^−2^), and 4-androsten-3beta,17beta-diol disulfate 2 (OR 0.461, 95% CI 0.250-0.851, *P* = 1.34 × 10^−2^) were found to be protective. In the carbohydrate category, erythrose served as a protective factor (OR 0.366, 95% CI 0.155-0.863, *P* = 2.16 × 10^−2^). Xenobiotics including glycerol 2-phosphate (OR 0.549, 95% CI 0.314-0.961, *P* = 3.58 × 10^−2^), 3-methylxanthine (OR 0.576, 95% CI 0.351-0.945, *P* = 2.91 × 10^−2^), and 4-ethylphenylsulfate (OR 0.580, 95% CI 0.361-0.931, *P* = 2.41 × 10^−2^) were identified as protective. A peptide, phenylalanylphenylalanine, was discovered to be a risk factor (OR 3.658, 95% CI 1.098-12.187, *P* = 3.47 × 10^−2^). The MR power calculation results revealed that our study had sufficient statistical power to detect the causal effect of these metabolites on GD. These findings provide insights into the relationship between GD and various metabolites across different pathways.

Given the possible concerns about the confounding effects of thyroid hormone levels on 10 particular metabolites, we meticulously analyzed potential confounders. After excluding confounding factors related to thyroid hormone levels, only kynurenine was found to be significantly influenced by such confounders. The listed confounders included self-reported hypothyroidism or myxedema, treatment with levothyroxine sodium, hypothyroidism, and treatment with thyroxine product, among others (Table S6 [21]). Using the IVW method, it was associated with an increased risk of GD (OR 2.700, 95% CI 1.224-5.969, *P* = 1.39 × 10^−2^) (Table S7 [21]). Notably, when these confounders were accounted for in our analysis, the significance of the kynurenine association persisted, suggesting the robustness of our findings.

### Evaluating Genetic Influence on GD and Metabolites

In our LDSC analyses exploring genetic influence on various metabolites, significant heritability was identified for a subset. Notably, kynurenine displayed a high heritability with an h^2^ value of 0.580 (*P* = 5.97 × 10^−16^), and glycerol 2-phosphate also showed substantial genetic influence, with an h^2^ of 0.353 (*P* = 2.13 × 10^−5^). 4-Androsten-3beta,17beta-diol disulfate 2 further evidenced significant SNP heritability with an h^2^ of 0.152 (*P* = 1.67 × 10^−2^). The rest of the metabolites, including those with NA values such as taurochenodeoxycholate, did not show statistically significant heritability (Table S8 [21]).

In the next phase of our investigation, we explored the genetic link between GD and 3 heritable metabolites: kynurenine, glycerol 2-phosphate, and 4-androsten-3beta,17beta-diol disulfate 2 (Table S9 [21]). Interestingly, no significant genetic linkage with GD was found (*P* > .05), suggesting that confounding effects from shared genetics on our MR assessments are unlikely. This strengthens the integrity of our MR evaluations and indicates that the relationships between these metabolites and GD are less likely to be influenced by shared genetic origins.

**Figure 4.**
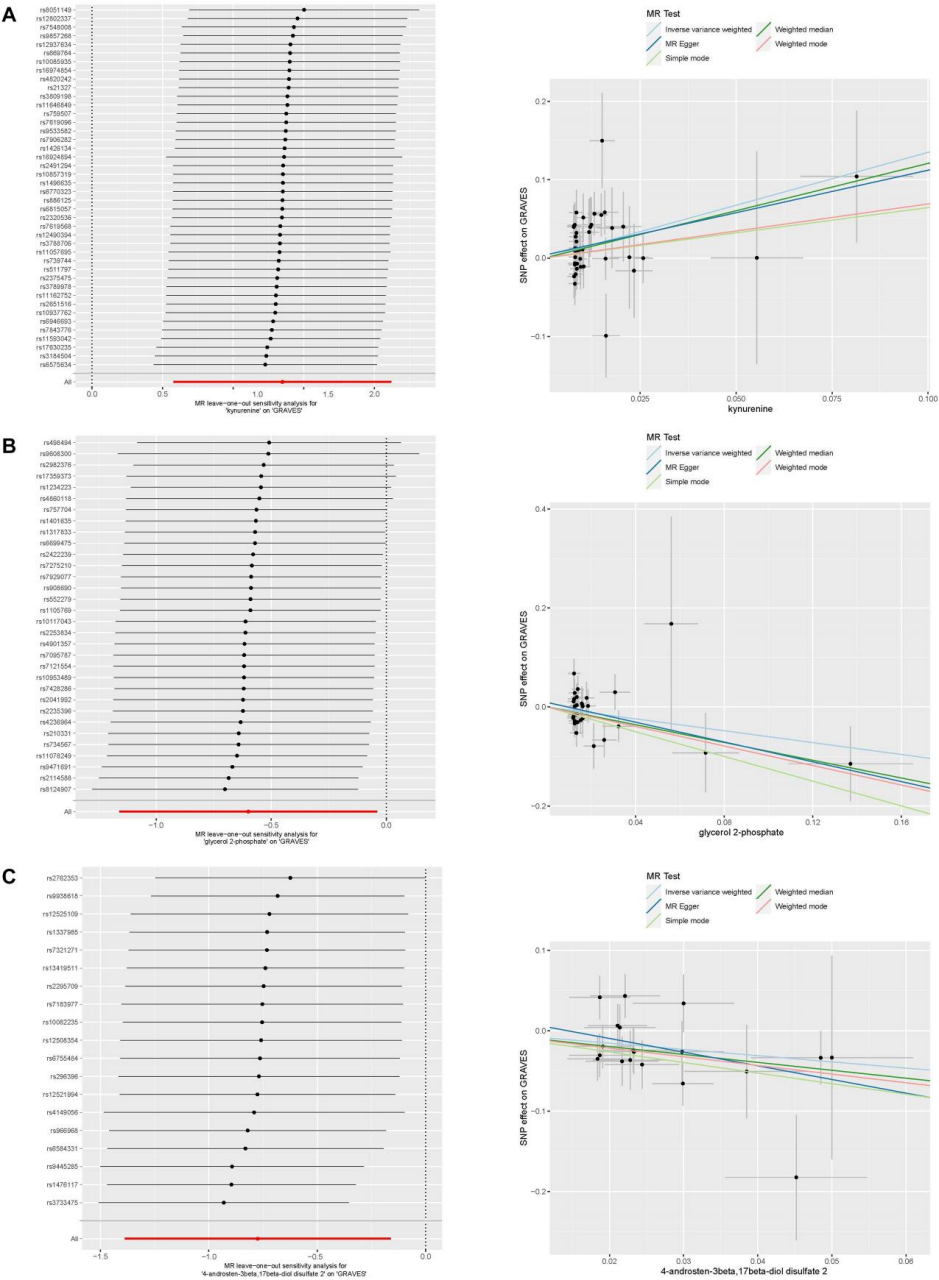
Scatter diagrams and leave-1-out analyses for 4 crucial metabolites pertaining to Graves disease: (A) kynurenine, (B) glycerol 2-phosphate, (C) 4-androsten-3beta,17beta-diol disulfate 2. In parts (A-C), the left panel unveils the leave-1-out evaluation, accentuating the stability of the causal estimate following singular exclusion of each instrumental SNP, while the right panel presents scatter diagrams of SNP exposure consequences vs SNP outcome consequences, with individual points denoting an instrumental SNP.

To further substantiate the causality between 3 metabolites and GD, a leave-1-out analysis was undertaken. The findings conclusively signaled that the associations were not the result of a singular SNP, but rather the collective influence of numerous SNPs (Fig. 4). Furthermore, the consequences of each sensitivity assessment were visually portrayed via scatter plots (Fig. 4).

## Discussion

In this comprehensive MR study, we utilized metabolomic information from 2 substantial European cohorts to scrutinize the genetic intertwining between serum metabolites and the predisposition to GD [18, 22]. The rigorous methodology we employed incorporated the exacting selection of IVs specifically adapted for MR analyses. This approach enabled an unswerving evaluation of the effect of individual metabolites on GD. Notably, this represents an avant-garde MR investigation that integrates genomics with metabolomics to pinpoint the causal connections between serum metabolites and GD.

Our research meticulously examines the nuanced interconnections between diverse metabolites and the susceptibility to GD, offering profound insights into potential foundational mechanisms. By employing the IVW methodology, we discerned 19 metabolites manifesting a pronounced association with the risk of GD [14]. Among these, 10 established metabolites maintained their noteworthy correlations after stringent adjustments for confounding factors and rigorous sensitivity assessments. To fortify the authenticity of our discoveries, mitigating the influence of shared genetic determinants that could obscure genuine associations, we implemented the LDSC analysis [15, 16]. This pivotal measure enabled us to ascertain that 3 metabolites (namely kynurenine, glycerol 2-phosphate, and 4-androsten-3beta,17beta-diol disulfate 2) demonstrated significant heritability. Crucially, these triad metabolites manifested no shared genetic interrelation with GD, amplifying our conviction in their potential causative significance.

In our MR study, significant associations between GD and several metabolites were discovered, enhancing our understanding of the underlying mechanisms. The amino acid kynurenine showed a profound positive association with GD, supported by highly significant genetic heritability. The role of the kynurenine pathway in inflammatory disorders has been well-documented, with kynurenine acting as an essential intermediary metabolite [33]. This pathway is initiated by the conversion of tryptophan to kynurenine, facilitated by indoleamine 2,3-dioxygenase, leading to further degradation into other kynurenines [34]. Within the context of GD, a disorder marked by an increased Th1 immune response and interferon-γ activity, the kynurenine pathway is evidently activated, with elevated Kyn levels reported in several inflammatory conditions [35-37]. Drawing from these findings, our research explores the genetic predisposition of these metabolic changes, specifically through a Mendelian inheritance perspective. While the activity of the kynurenine pathway is observed in GD [11, 33], the exact genetic mechanisms remain largely unexplored. This is particularly intriguing considering the variations in serum levels of neopterin, KTR, and kynurenines in GD [38-40]. Our study introduces a genetic framework to these observations, suggesting that these metabolic alterations might follow a Mendelian pattern. Notably, elevated Kyn levels and suppressed Pic in patients with GD hint at a genetic underpinning, potentially explaining the discrepancies observed in previous research [39, 40]. The exploration of the kynurenine pathway in the context of Mendelian genetics offers a novel perspective, possibly leading to a more nuanced understanding the complexity of GD. However, this is still a speculative interpretation, and rigorous experimental validation is required to establish the genetic foundation of these metabolic shifts conclusively.

In the broader context of our study, the associations identified with specific metabolites offer significant insights into the etiology of GD. Notably, glycerol 2-phosphate, a xenobiotic, exhibited a negative association with GD. This connection may imply that environmental exposures exert a profound effect on the metabolic landscape of GD, thereby influencing disease risk. Xenobiotics, often associated with external influences such as diet or pollutants, may modulate metabolic pathways [41], reflecting an unexplored dimension in the multifaceted nature of GD. Similarly, the lipid metabolite 4-androsten-3beta,17beta-diol disulfate 2 showed a negative association. Given the known role of lipids in immune response and inflammation [42], this finding aligns with the known pathophysiology of GD [1]. It is plausible that specific lipid metabolites might be tied to inflammatory pathways in GD, underscoring a potential target for intervention or diagnosis. Collectively, these associations illustrate a complex interplay between genetic factors, environmental exposures, and metabolic processes in the etiology of GD. Supported by significant IVW statistics and LDSC genetic heritability, these results not only contribute to our understanding of the underlying mechanisms of GD but also pave the way for novel, targeted therapeutic strategies.

Our study, while providing insights into the metabolic underpinnings of GD, has limitations. Primarily, the FinnGen cohort's modest number of patients with GD may affect the robustness our findings. Moreover, the low variance in serum metabolite levels explained by the IVs (0.002-0.708%) suggests potential weak instrument bias, potentially affecting causal inference precision. Consequently, these results warrant cautious interpretation. Furthermore, our findings may be less applicable to non-Northern European populations due to genetic and environmental differences. Additionally, the relationships identified largely rely on statistical correlations, underscoring the need for experimental validation. Lastly, our LDSC analysis may have been hampered by factors like insufficient SNP representation or data integrity concerns. Given current database access, we earnestly anticipate future research advancements in GD complications, notably Graves orbitopathy.

Our study illuminates the multifaceted relationship between metabolites and GD, revealing protective effects of glycerol 2-phosphate and 4-androsten-3beta,17beta-diol disulfate 2, and risk factors in kynurenine. These insights lay the groundwork for future exploration, possibly leading to innovative prevention, early detection, or personalized treatments for GD. Understanding these nuanced connections may unveil novel strategies targeting this complex and prevalent condition.

## Data Availability

The original contributions presented in the study are included in the article/supplementary material. Further inquiries can be directed to the corresponding author.

## Abbreviations

GD: Graves disease
GWAS: genome-wide association study
IV: instrumental variable
IVW: inverse variance weighted
KEGG: Kyoto Encyclopedia of Genes and Genomes
MR: Mendelian randomization
LDSC: linkage disequilibrium score regression
SNP: single nucleotide polymorphism
